# Anisotropic layered Bi_2_Te_3_-In_2_Te_3_ composites: control of interface density for tuning of thermoelectric properties

**DOI:** 10.1038/srep43611

**Published:** 2017-03-08

**Authors:** Dongmei Liu, Xinzhong Li, Pedro Miguel de Castro Borlido, Silvana Botti, Roland Schmechel, Markus Rettenmayr

**Affiliations:** 1 Otto Schott Institute of Materials Research, Friedrich Schiller University Jena, Löbdergraben 32, D-07743 Jena, Germany; 2 School of Materials Science and Engineering, Harbin Institute of Technology, West Da-Zhi Street 92, 150001 Harbin, China; 3 Institute for Condensed Matter Theory and Optics, Friedrich Schiller University Jena, Max-Wien-Platz 1, 07743 Jena, Germany; 4 Institute of Technology for Nanostructures and CENIDE, University of Duisburg-Essen, Bismarkstraße 81, 47057 Duisburg, Germany; 5 Center for Energy and Environmental Chemistry, Philosophenweg 7, D-07743 Jena, Germany

## Abstract

Layered (Bi_1−x_In_x_)_2_Te_3_-In_2_Te_3_ (x = 0.075) composites of pronounced anisotropy in structure and thermoelectric properties were produced by zone melting and subsequent coherent precipitation of In_2_Te_3_ from a (Bi_1−x_In_x_)_2_Te_3_ (x > 0.075) matrix. Employing solid state phase transformation, the Bi_2_Te_3_/In_2_Te_3_ interface density was tuned by modifying the driving force for In_2_Te_3_ precipitation. The structure-property relationship in this strongly anisotropic material is characterized thoroughly and systematically for the first time. Unexpectedly, with increasing Bi_2_Te_3_/In_2_Te_3_ interface density, an increase in electrical conductivity and a decrease in the absolute Seebeck coefficient were found. This is likely to be due to electron accumulation layers at the Bi_2_Te_3_/In_2_Te_3_ interfaces and the interplay of bipolar transport in Bi_2_Te_3_. Significantly improved thermoelectric properties of Bi_2_Te_3_-In_2_Te_3_ composites as compared to the single phase (Bi_1−x_In_x_)_2_Te_3_ solid solution are obtained.

Research on thermoelectrics has witnessed a renewal of interest with the drastic improvement of thermoelectric properties owing to nanoscaled structures, e.g. in thin film superlattice/quantum well multilayered structures^1^,^2^,^3^. Although the drawbacks of thin film materials such as their small scale, high cost and processing difficulties restrict their practical application, their favorable properties have stimulated extensive research on preparing nanostructured materials by bulk processes. Examples are high temperature high pressure pressing or spark plasma sintering of nanoscale powder^4^,^5^. Nanostructured materials prepared by bulk processes, which either contain nanometer sized grains or nanoscale precipitates, have been shown to exhibit a strongly enhanced thermoelectric performance. It is widely accepted that the enhancement is due to a high density of interfaces such as grain boundaries and heterophase interfaces^6^,^7^,^8^,^9^. Under certain conditions, the interfaces in thermoelectric materials augmented the Seebeck coefficient and reduced thermal and electrical conductivity by energy filtering of charged carriers^10^. Interfaces have on the other hand been found to induce an increase in electrical conductivity by selective scattering of lower mobility charged carriers by interfacial charged defects^11^. The questions how the interface density on the nanoscale can be controlled and how it affects the transport properties are challenging from both the engineering and the scientific viewpoint.

Several traditional metallurgical methods have shown their potential to achieve nanoscaled structures and to generate interfaces that are beneficial for the thermoelectric properties^12^,^13^,^14^,^15^,^16^,^17^,^18^,^19^,^20^,^21^. Physical processes such as eutectic reaction^12^,^13^,^14^,^15^, solid state precipitation^16^,^17^,^18^,^19^, and spinodal decomposition^20^,^21^ have been exploited favorably and reproducibly. For clarifying structure-property relationships, the microstructural length scales can be varied systematically in a wide range by adjusting the processing parameters. For example, the lamellar spacing of the layered structure of PbTe and Sb_2_Te_3_ formed by the decomposition of Pb_2_Sb_6_Te_11_ is controlled by the temperature and time of the decomposition process^16^ and has been found to range from 30 nm at 200 °C to 200 nm at 500 °C. Up to present, the characterization of thermoelectric properties of lamellar thermoelectric materials generated by solidification/solid state phase transformation is either utterly lacking or carried out for isotropic materials prepared by conventional solidification processes. Recalling that lamellar structures formed via liquid/solid or solid state phase transformations exhibit preferential crystallographic orientations with respect to each other, a systematic analysis of the structure-property relationships should include the effects of anisotropy, and precise control of the lamellar growth direction should be attained in such materials, as e.g. by employing directional solidification techniques. In the present work, we apply a specially developed zone melting technique on Bi_2_Te_3_-In_2_Te_3_ samples, controlling the crystal orientation of the parent solid solution and the lamellar structure that is formed by solid state precipitation. The lamellar spacing is adjusted in a wide range by varying the supersaturation of the parent solid solution. Thoroughly characterized microstructural parameters are correlated with the thermoelectric performance for the first time. Suppression of bipolar transport and enhanced interface density contributed to an improved performance of the material.

## Experimental

### Synthesis

**Figure 1:**
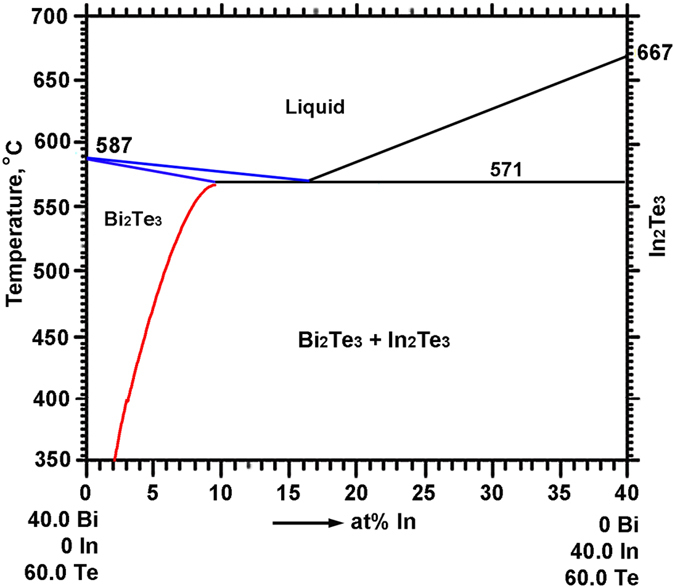
Pseudo-binary Bi_2_Te_3_-In_2_Te_3_ phase diagram^17^,^22^,^23^. The blue lines illustrate the recently reported solidus and liquidus lines^22^, and the red line illustrates the recently reported solvus line of In in Bi_2_Te_3_ from ref. 17.

Oriented (Bi,In)_2_Te_3_ (containing 3, 4, 6, and 7.5 at%In) solid solutions with the {001} plane being parallel to the growth axis were obtained by a seeding zone melting technique^22^. This technique yields an especially uniform composition over the entire length of the zone melted region along the growth direction. More details on the control of macroscopic homogeneity via seeding zone melting can be found in ref. 22. Macroscopic homogeneity is a necessary precondition for the homogeneous distribution of precipitates and thus for a uniform thermoelectric performance of the bulk composite samples. The driving force for precipitation is temperature and concentration dependent, as can bee directly seen in the pseudo-binary Bi_2_Te_3_-In_2_Te_3_ phase diagram according to previous work in the literature^17^,^23^ and our present work (Fig. 1). The blue lines illustrate the recently reported solidus and liquidus lines^22^, based on which the concentration of the seed alloy for the seeding zone melting is selected. The seed alloys for 3, 4, 6 and 7.5 at% are 7, 9, 12.5 and 13.5 at%In, respectively.

Samples with In concentrations of 4, 6 and 7.5 at% were vacuum sealed in quartz and isothermally annealed for the precipitation of In_2_Te_3_. Note that homogenization after seeding zone melting is not necessary, as a perfectly homogeneous crystal is generated. This reduces possible artifacts and the misinterpretation of measured values significantly. Considering the temperature dependent solubility of In in Bi_2_Te_3_, as illustrated by the red line in Fig. 1, a uniform annealing temperature (400 °C in the present work) was chosen for all samples, resulting in a uniform and identical In concentration in the Bi_2_Te_3_ matrix in all samples, but a different Bi_2_Te_3_/In_2_Te_3_ interface density. After annealing for 6 days, the sample was water quenched. Annealing for a longer time of up to 12 days was also performed, but no further change in microstructure and thermoelectric properties was observed. This confirms that 6 days are long enough to reach the equilibrium. The microstructure consists of micro-/nanoscaled **B**i_2_**T**e_3_/**I**n_2_**T**e_3_ (“BTIT”) lamellae of different spacing, depending on the initial In concentration. For simplification, all the BTIT composites are termed as BTIT-c with the initial In concentration c = 4, 6, 7.5, in at%In.

### Materials characterization

The samples were prepared metallographically by grinding with a series of SiC papers up to a grit size of 4000, by polishing with 3 μm and 1 μm Al_2_O_3_ powder suspension, and finally polishing with 50 nm colloidal silica. The microstructure was observed using a scanning electron microscope (SEM) equipped with a backscattered electron (BSE) detector and energy dispersive X-ray (EDX) spectrometry. Thickness and volume fraction of the phases in the samples were analyzed using the image processing software Image J. X-ray diffraction (XRD) was used to characterize the constituent phase(s) of the sample. Thin sections of the specimens were examined in the transmission electron microscope (TEM, JEM 3010 - HR pole piece, JEOL). TEM samples were prepared by means of Focused Ion Beam (FIB) using an *in-situ* lift-out technique.

Measurement of thermoelectric properties was performed for all samples. Plates of 4 × 4 × 10 mm^2^ and ∅6 × 1 mm^2^ were cut along and perpendicular to the growth direction, respectively. The electrical conductivity (σ) and Seebeck coefficient (*S*) were measured using the thermoelectric measurement system ZEM-3 in the temperature range from room temperature to 300 °C. The equilibrium phase diagram^17^ suggests that the temperature dependent solubility may lead to microstructural changes during aforementioned measurement of the thermoelectric properties. For assuring the accumulated annealing effect (heating rate 1.5 K/min) during measurement on microstructure and properties, repeated measurements on one sample were performed. The measured properties during the second cycle are essentially the same as the first time, indicating the negligible changes within the measurement time. The thermal conductivity (*k*_tot_) was calculated from the values of thermal diffusivity (*α*), density (ρ) and specific heat (C_P_) by the relationship *k* = *α*ρC_P_, where *α* was measured using a laser flash system (Netzsch LFA-457), ρ was measured by the Archimedes method, and C_P_ was determined by a combination of the Dulong-Petit law and a differential scanning calorimeter (Netzsch STA-449FA). The measured values fall into the same range and are also consistent with the calculated values by the Dulong-Petit law. For each sample state, 3 samples prepared by the same experimental procedure were characterized with respect to their thermoelectric properties. All samples exhibited similar or identical temperature dependent Seebeck coefficients and thermal diffusivities. A maximum variation of ±12% of the measured electrical conductivity was observed, and average values were finally used in the present work. The variation of the measured heat capacities and thermal conductivities was ±5%. The electronic contribution to the thermal conductivity (*k*_e_) is determined from the electrical conductivity utilizing the Wiedemann-Franz law, *k*_e_ = *LσT*. From the total thermal conductivity *k*_tot_, the electronic contribution (*k*_e_) is subtracted, yielding *k*_LB_, the lattice thermal conductivity *k*_L_ plus further contributions, particularly the bipolar contribution *k*_B_.

## Results and Discussion

### Strong anisotropy in structure and thermoelectric properties of layered Bi_2_Te_3_-In_2_Te_3_

**Figure 2:**
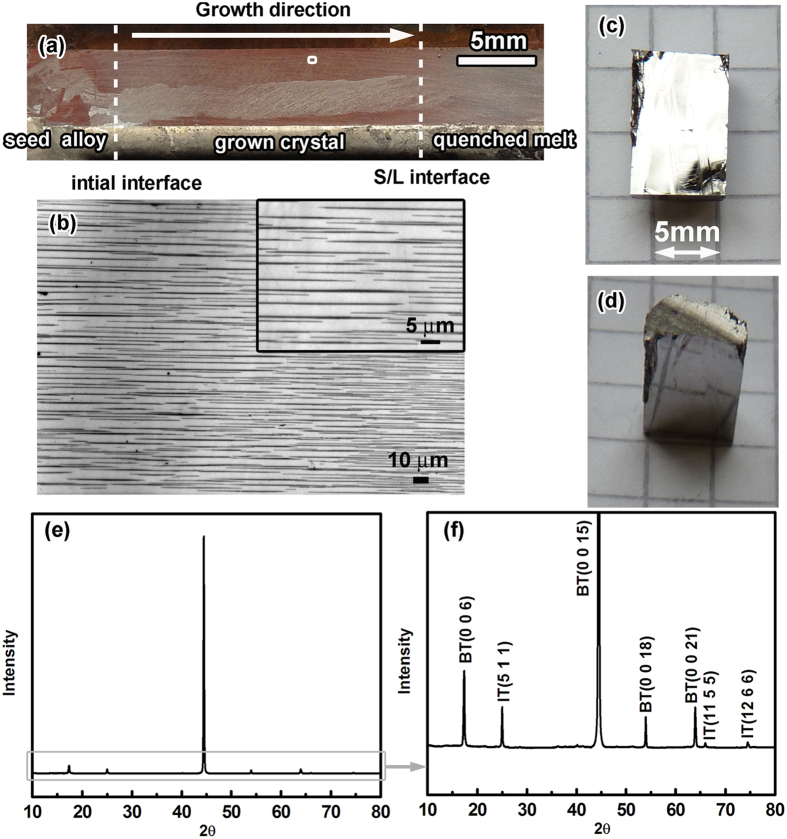
(**a**) Typical macroscopic structure of the Bi-In-Te samples; (**b**) BTIT lamellar structure in a longitudinal section, where the bright phase represents Bi_2_Te_3_ and the dark phase represents In_2_Te_3_; (**c,d**) cleaved interface; (**e,f**) XRD patterns.

Figure 2 shows a typical morphology and the corresponding XRD pattern of an ingot after annealing. Over the width of the ingot, there are only a few grains (see Fig. 2(a)), all of them with the (001) plane parallel to the growth axis (see XRD patterns in Fig. 2e,f). The lamellar structures obtained after annealing also exhibit a preferential orientation of the lamellae along the growth direction, as shown in Fig. 2(b). The solidified sample is very sensitive to cleavage along the axis during the cutting process, as shown in Fig. 2(c,d), demonstrating the pronounced crystallographic anisotropy of the samples. The XRD pattern (Fig. 2(e,f)) confirm that the the Bi_2_Te_3_ grains grow in a preferential orientation perpendicular to the [001] direction. In_2_Te_3_ peaks also show the anisotropy, considering that all the visible peaks are indexed (*h k l*) with *k* = *l*.

**Figure 3:**
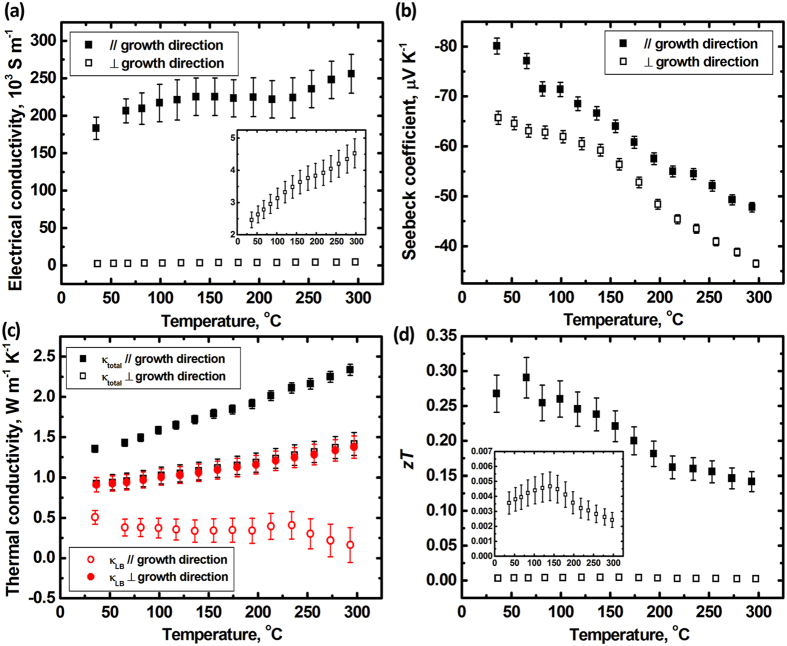
Temperature dependence of (**a**) electrical conductivity σ, (**b**) Seebeck coefficient *S*, (**c**) thermal conductivity *k*, and (**d**) *zT* for BTIT-7.5 in the directions parallel (//) and perpendicular (⊥) to the crystal growth direction. Solid symbols are for parallel (//), open symbols for perpendicular (⊥).

Since the samples produced by zone melting and precipitation exhibit pronounced microstructural anisotropy, also anisotropic thermoelectric properties are expected. Figure 3 shows the temperature dependence of the thermoelectric properties of the sample of which the initial In concentration is 7.5 at%In, i.e. BTIT-7.5, for measurement directions parallel (//) and perpendicular (⊥) to the crystal growth direction. The electrical conductivity σ_//_ is nearly two orders of magnitude higher than σ_⊥_ for the whole temperature range (Fig. 3(a)). This anisotropy in conductivity is dramatically more pronounced than that generally expected for anisotropic single phase Bi_2_Te_3_, where the electrical conductivity σ_//_ is only 3…7 times larger than σ_⊥_^24^,^25^,^26^. For the Seebeck coefficient *S*, The negative values agree with the n-type transport behavior of Bi_2_Te_3_^27^,^28^. The decrease of |*S*|_//_ with temperature has been assigned to mixed conduction of holes and electrons (bipolar conduction) in this temperature range due to the increasing number of thermally generated holes, considering the narrow band gap of (or below) 130 meV^29^. The bipolar contribution is also the reason for the increasing conductivity with increasing temperature (3a). As opposed to the strong anisotropy in the electrical conductivity, |*S*|_⊥_ is only a little smaller than |*S*|_//_ and exhibits similar temperature dependence. The bright phase in Fig. 2(b) represents the (Bi, In)_2_Te_3_ phase, whose In concentration was measured as ~3 at%, and the black phase represents the In_2_Te_3_ phase with negligible Bi concentration. More details on the composition distribution across the two phases will be discussed in detail below.

The temperature dependence of the thermal conductivities in both directions is shown in Fig. 3(c). The thermal conductivity in the direction parallel to the growth direction *k*_tot//_ is higher than *k*_tot⊥_, reflecting also the anisotropy of the electrical conductivity. However, after subtracting the electronic contribution (*k*_e_ = *LσT*) from the total thermal conductivity, the remaining thermal conductivity *k*_LB//_ becomes smaller than *k*_LB⊥_. The value of the Lorenz number is not only dependent on charged carrier concentration and temperature, but also can be influenced by quantum well effects, which e.g. occurs in superlattice/quantum well thin film materials^30^,^31^. An accurate determination of the Lorenz number of all BTIT composites would require considerably more effort,i.e. the determination of temperature dependent charged carrier concentration, effective carrier mass, Fermi level and so on. For the BTIT composites in the present work, the existence of both electrons and holes, especially the anisotropy not only from the Bi_2_Te_3_ phase but also from the lamellar aligned microstructure, make the determination of the charge carrier concentration a complex issue. In the literature, Lorenz numbers in the range of 1.2…1.4 × 10^−8^ 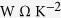 for a Bi_2_Te_3_-based superlattice^32^ to 1.6…2.0 × 10^−8^ 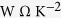 for single-phase Bi_2_Te_3_^33^,^34^,^35^ have been reported. The value 1.6 × 10^−8^ 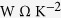, which is mostly used for composite materials^3^,^33^,^34^,^35^ and is also used in the present work. Besides, Lorenz numbers calculated with the equation: 
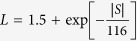
 and ranging from 1.8 to 2.2 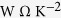 were also tried out for the calculation of *k*_LB_. However, this lead to non-physical negative *k*_LB//_ values of BTIT-7.5, most likely due to the complex band structure or the complex scattering mechanism in BTIT. According to published work on Bi_2_Te_3_^36^, the pure lattice thermal conductivity in the given temperature range should be inversely proportional to *T* and thus decrease with temperature. Whereas this may approximately hold for *k*_LB//_, it is definitively not the case for *k*_LB⊥_, indicating a bipolar contribution to the thermal conductivity (see discussion below). The figure of merit, 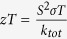, in the direction perpendicular to the growth direction is drastically smaller than in the direction parallel to the growth direction, despite the lower thermal conductivity. This is mainly due to the drastically lower σ_⊥_, as shown in Fig. 3(a).

### Analytical models of the effective properties of layered Bi_2_Te_3_-In_2_Te_3_

When discussing the thermoelectric properties of BTIT, one has to keep in mind that the material is a two-phase composite material (see Fig. 2). Effective medium theory (EMT) and its generalized derivation (GEMT)^37^,^38^,^39^ have been used to predict the thermoelectric properties of composite materials where a randomly distributed secondary phase is embedded in a matrix^40^,^41^. As to BTIT-7.5 in the present work, strong anisotropy effects in structure and thermoelectric properties were confirmed. Hence, it is most appropriate to use a parallel model^42^,^43^ to predict the transport properties along the layer orientation, and a series model^42^,^43^ for the properties perpendicular to the layer orientation. It is worth noting that effects of the interface density are not considered in all these models, which will be shown to be an essential limitation later in the discussion section.

The total Seebeck coefficient (*S*) as calculated by a mixture rule follows from the Seebeck coefficients of the two phases 1 and 2 by one of the relationships


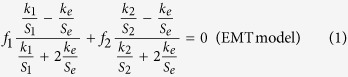



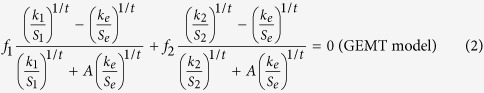



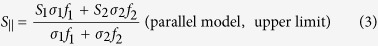



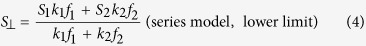


and the total electrical conductivity (*σ*) should be a consequence of the conductivity of the individual phases following


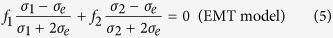



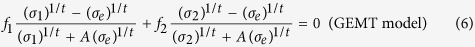



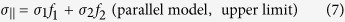



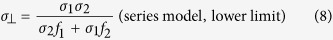


where *f* is the volume fraction and *k* is the total thermal conductivity of the respective constituent phase, A is a constant that depends on the actual percolation threshold 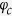 (of phase 2 in phase 1) through the equation 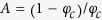, *t* is a constant representing the asymmetry of the microstructure, and the subscripts 1, 2 and *e* refer to Bi_2_Te_3_, In_2_Te_3_ and BTIT, respectively^37^,^38^,^39^.

Irrespective of the chosen model, the calculated properties of a composite cannot exceed those of one of the constituents. The two phases in our material are substantially different in their thermoelectric properties. Bi_2_Te_3_ with dissolved In is known to be n-type material for In concentrations ranging from 2…6.5 at%^27^,^28^, with a conductivity in the order of 10^4^ Ω^−1^m^−1^, In_2_Te_3_ is a p-type material with a very low conductivity in the order of 10^−5^ Ω^−1^m^−1^ 44,45,46. Since the In concentration in the Bi_2_Te_3_ layers of BTIT is ~3at%, the experimental room temperature property values for single phase Bi_2_Te_3_ with 3at%In, i.e. BT-3, are used for the calculation. A Seebeck coefficient of −114 μV K^−1^, an electrical conductivity of 33 × 10^3^ S m^−1^, and a thermal conductivity of 1.36 W m^−1^ K^−1^ are used for the calculation. Since the In_2_Te_3_ phase in BTIT samples is plate-like with thicknesses of 100…200 nm, the room temperature thermoelectric properties for In_2_Te_3_ are taken from literature on In_2_Te_3_ films^44^,^45^,^46^. The Seebeck coefficient for In_2_Te_3_ is set to 185 μV K^−1^ 44, the electrical conductivity to 5.6 × 10^−3^ S m^−1^ 45, and the thermal conductivity to 1.1 W m^−1^ K^−1^ 46.

When using the EMT or GEMT models to calculate the Seebeck coefficient of BTIT, equations (1) and (2) listed above do not work. The reason is that Bi_2_Te_3_ is an n-type material with negative Seebeck coefficient, and In_2_Te_3_ is a p-type material with a positive Seebeck coefficient. Considering the opposite contribution of Bi_2_Te_3_ and In_2_Te_3_ to the total Seebeck coefficient of the composite, the EMT/GEMT models need to be adapted, as shown below:


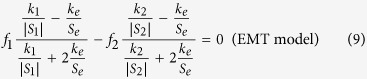



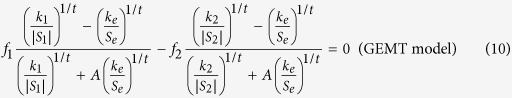


**Figure 4:**
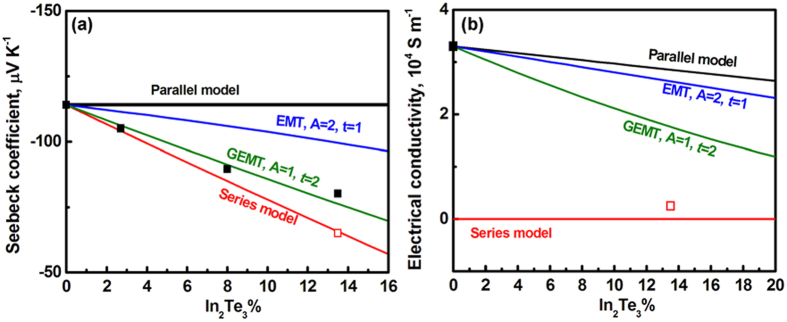
Calculated dependence of room temperature (**a**) Seebeck coefficient and (**b**) electrical conductivity of BTIT on the volume fraction of In_2_Te_3_.

The calculation results for the properties of the BTIT composites at room temperature are plotted in Fig. 4 together with the measured values. In the direction perpendicular to the growth direction, the measured properties of BTIT-7.5 are very close to the calculations with the series model. The electrical conductivity in the perpendicular orientation is dominated by In_2_Te_3_ phase, see eq. (8), i.e. σ_⊥_≈*σ*_In2Te3_. BTIT-7.5 exhibits such a low electrical conductivity in the direction perpendicular to the growth direction (Fig. 3(a)). Due to the anisotropic microstructure, the transport properties along the growth direction should follow the parallel model and thus be dominated by the properties of Bi_2_Te_3_, see eq. (7), i.e. *σ*_//_ ≈ *σ*_Bi2Te3_ and eq. (3) *S*_//_ ≈ *S*_Bi2Te3_. However, the experimentally measured thermoelectric properties in the parallel direction (both σ_//_ and *S*_//_) of BTIT-7.5 divert drastically from the calculated values. The measured *S*_//_ values are close to the values calculated with the GEMT model (A = 1, *t* = 2). All models suggest a decreasing σ_//_ with increasing volume fraction of In_2_Te_3_. However, the large difference between the calculated σ and σ_//_ suggest that the Bi_2_Te_3_/In_2_Te_3_ interfaces influence the thermoelectric properties in a more complex manner.

### Tuning of interface density in Bi_2_Te_3_-In_2_Te_3_ via solid state phase transformation

**Figure 5:**
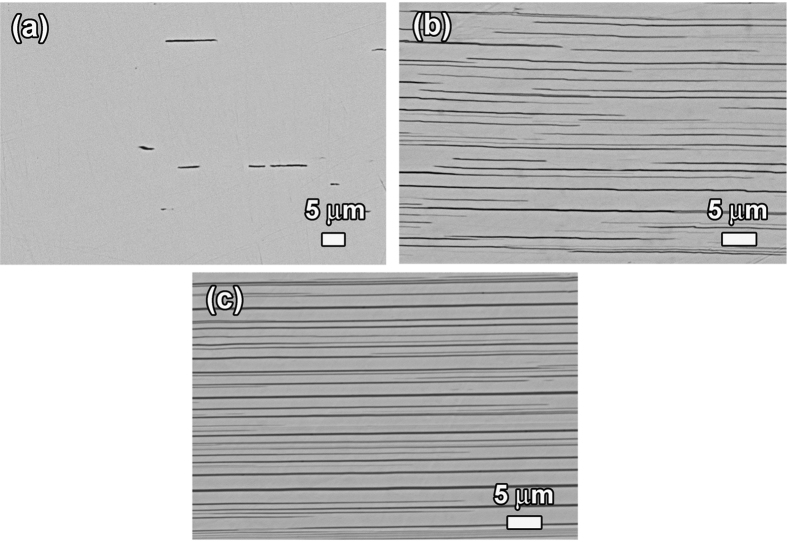
Microstructure of BTIT-c (c = 4, 6, 7.5 at% In), with the dark phase representing In_2_Te_3_, and the grey phase representing (Bi,In)_2_Te_3_ (~3at%In); (**a**) 4 at%In; (**b**) 6 at%In; (**c**) 7.5 at%In.

**Figure 6:**
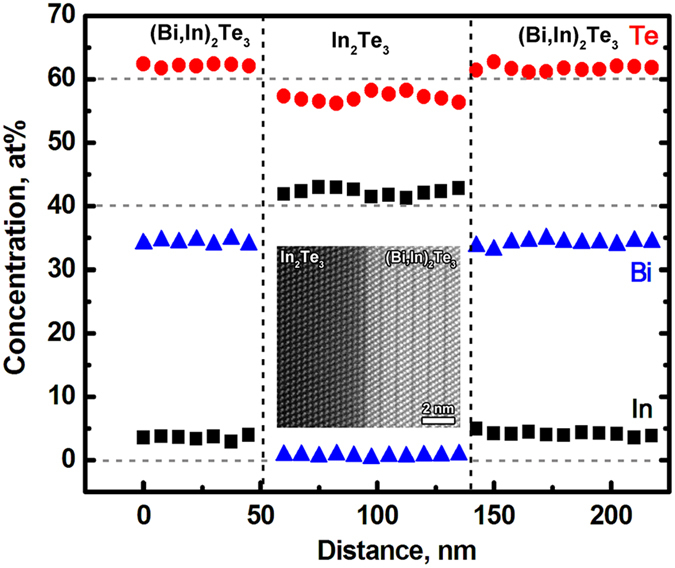
EDX composition analysis in the TEM across (Bi, In)_2_Te_3_ and In_2_Te_3_ layers; the HRTEM image in the inset illustrates the sharp (Bi, In)_2_Te_3_/In_2_Te_3_ interface.

To get deeper insight into the effect of the Bi_2_Te_3_/In_2_Te_3_ interfaces on the transport behavior of BTIT, the interface density in BTIT was varied in a controlled way. Since the thermoelectric properties of Bi_2_Te_3_ are very sensitive to the composition, the Bi_2_Te_3_/In_2_Te_3_ interface density was modified by changing the supersaturation of the parent (Bi, In)_2_Te_3_ phase while maintaining the same annealing temperature, i.e. the same composition of each constituent phase. Figure 5 shows cross section microstructures of (Bi, In)_2_Te_3_ (4, 6, and 7.5 at%In) after precipitation annealing at 400 °C. For avoiding projection effects in the lamellar spacing measurements, microstructural analysis was performed in cross sections of the samples. The grey phase in Fig. 5 represents the (Bi, In)_2_Te_3_ phase with an identical In concentration of ~3 at% for all samples, and the black phase represents the In_2_Te_3_ phase with negligible Bi content. The composition distribution in each phase was determined in the TEM, as shown in Fig. 6. In each phase, the concentration of each element is homogeneous within the measurement scatter of EDX. Interestingly, a slight but significant variation in the Te concentration across Bi_2_Te_3_/In_2_Te_3_ interfaces is observed. The concentration of Te in the (Bi, In)_2_Te_3_ phase is a little higher than 60 at%, while the Te concentration in the In_2_Te_3_ phase is somewhat lower than 60 at%. Inhomogeneity and lattice misfit at heterophase interfaces have been demonstrated to cause transport processes across the interface and hence change the thermoelectric performance. A high resolution TEM image of a Bi_2_Te_3_/In_2_Te_3_ interface is also shown in Fig. 6. Together with the EDX analysis, this confirms that there is no diffusion layer and that the Bi_2_Te_3_/In_2_Te_3_ interface is sharp and coherent. As can be clearly seen from Fig. 5, the volume fraction of In_2_Te_3_ increases and the lamellar spacing decreases with increasing In concentration. Simultaneously, the length of the In_2_Te_3_ plates increases. For all samples, the thickness of the In_2_Te_3_ plates is similar, in the order of ~100 to 200 nm. For BTIT-6, the lamellar spacing ranges from ~500 nm to ~3.5 μm, with the majority of lamellar spacings being in the size class ~1.2 μm. For BTIT-7.5, the lamellar spacing is smaller, ranging from ~200 nm to ~2 μm, and most of the lamellae have a spacing below 1 μm. For verifying that 6 days are enough for reaching thermodynamic equilibrium at 400 °C, we compared the measured lamellar spacing values with the diffusion length, 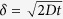, where *D* is the diffusion coefficient and *t* is the involved time scale. *D* is of the order of 10^−12^ m^2^ s^−1^, as found in previous work on the diffusion of metallic elements in Bi_2_Te_3_^47^,^48^,^49^. The calculated diffusion length in 6 days is 1440 μm, which is much larger than the observed spacing between neighboring In_2_Te_3_ precipitates for all BTIT samples. This in turn confirms that the equilibrium state at 400 °C is reached. With the In concentration increasing from 4 to 7.5 at%, the volume fraction of In_2_Te_3_ after annealing was measured as 1.5 ± 0.5%, 7.3 ± 1.5% and 13.5 ± 1.5%, respectively. These values are consistent with those calculated by the lever rule based on the solubility of In in Bi_2_Te_3_ (3at%In at 400 °C) and negligible solubility of Bi in In_2_Te_3_. In the present work, it can be seen from Fig. 5 that the increase in In_2_Te_3_ volume fraction mainly comes from the decrease of lamellar spacing rather than the increase in thickness of In_2_Te_3_.

### Correlation between the interface density and thermoelectric properties of Bi_2_Te_3_-In_2_Te_3_

**Figure 7:**
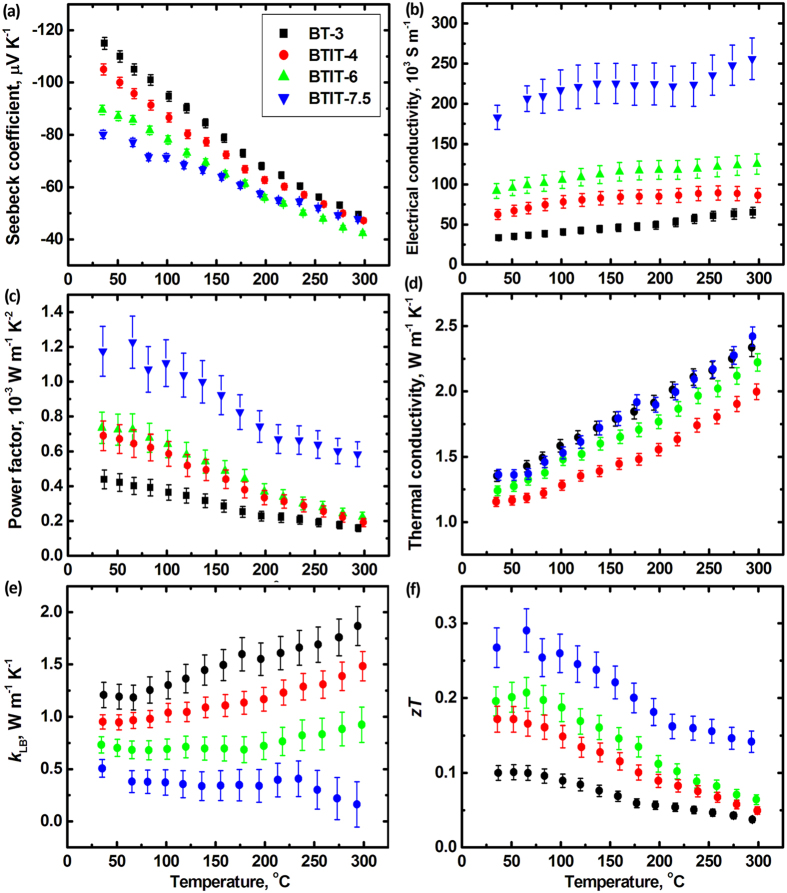
Temperature dependence of Seebeck coefficient *S* (**a**), electrical conductivity *σ* (**b**), power factor (**c**), thermal conductivity *k*_tot_ (**d**), *k*_tot_ − *k*_LB_ (**e**), and *zT* (**f**) of BT-3 and BTIT-4, -6, -7.5.

To get deeper insight into the physical transport mechanisms, the thermoelectric properties of the solid solution sample (BT-3 at%In) and the composite samples (BTIT-4, -6, -7.5 at%In) were characterized in the temperature range from room temperature to 300 °C, see Fig. 7. Since the thermoelectric properties perpendicular to the growth direction are expected to be technically uninteresting, as shown in Figs 3 and 4, only the properties parallel to the sample axis were studied. All samples exhibit a similar temperature dependence trend of the thermoelectric properties. Negative values of the Seebeck coefficient throughout the whole measured temperature range are found for all samples (Fig. 7(a)), indicating n-type transport behavior. With increasing temperature from room temperature to 300 °C, the absolute value of the Seebeck coefficient |*S*| decreases, and the electrical conductivity σ increases, indicating mixed conduction or bipolar transport behavior^29^. The BT-3 sample shows the highest |*S*|, but the lowest σ of all samples. With increasing volume fraction of In_2_Te_3_ and decreasing lamellar spacing, |*S*| decreases while σ increases. Since the conductivity of In_2_Te_3_ is much smaller than that of Bi_2_Te_3_, the increase of σ with increasing In_2_Te_3_ volume fraction is not straightforward to understand.

For qualitatively analyzing the possible reason for the increasing σ, Hall tests at room temperature were performed on all the samples. The measured Hall coefficients of BTIT at room temperature are given in Table 1. The carrier concentration can be calculated from R_H_ via the equation R_H_ = *A/ne*, where *A* is related to the anisotropy factor of the effective carrier mass and the Fermi energy levels^50^. However, the microstructure/property anisotropy and the existence of two types of material, p-type In_2_Te_3_ and n-type Bi_2_Te_3_, (i.e. the coexistence of both holes and electrons) make the accurate determination of *A* not straightforward. Due to a lack of detailed information, an approximation, R_H_ = *1/ne* is used in our present work. We get qualitative information concerning the increase of the electrical conductivity with increasing BT/IT interface density, as listed in Table 1. The measured carrier concentration does not differ much between the samples. This indicates that the contribution of In_2_Te_3_ and the BT/IT interfaces to the carrier concentration is negligible. This is partly due to the very low room temperature hole concentration of In_2_Te_3_, 7.8 × 10 ^9^cm^−3^, which is nearly 10 orders of magnitude lower than that of BT-3In. The increasing σ of BTIT with increasing In_2_Te_3_ volume fraction mainly comes from the increasing carrier mobility.

The total thermal conductivity shows a non-uniform trend: BTIT-4 and BTIT-6 exhibit a lower *k*_*tot*_ than BT-3, and BTIT-7.5 exhibits a comparable *k*_*tot*_ as BT-3, as shown in Fig. 7(d). After subtracting the electronic contribution, the thermal conductivity shows a clear trend in Fig. 7(e) – it distinctly decreases with increasing In_2_Te_3_ fraction. The remaining thermal conductivity after subtracting the electronic contribution contains the lattice thermal conductivity and the bipolar contribution. The pure lattice thermal conductivity should decrease with increasing temperature due to phonon-phonon interaction^36^. In contrast, the bipolar contribution increases due to the increasing number of electron/hole pairs. Thus, all samples show a significant bipolar contribution to the thermal conductivity (Fig. 7(e)), in agreement with the mixed conduction behavior of the Seebeck coefficient and the electrical conductivity. Nevertheless, k_LB_ decreases with increasing In_2_Te_3_ fraction, and for BTIT-7.5 the overall temperature dependence is inverted from increasing to decreasing (Fig. 7(e)). This indicates the suppression of the bipolar contribution by a decreasing BTIT lamellar spacing. Bipolar transport contributions are counterproductive for the thermoelectric properties, because they do not only increase the thermal conductivity, but also lower the Seebeck coefficient. Thus, BTIT-7.5 with the lowest bipolar contribution has the highest *zT* value, see Fig. 7(f).

### n-Bi_2_Te_3_/p-In_2_Te_3_ hetero-interface effects

The enhanced *zT* values of BTIT are mainly attributed to the increased electrical conductivity and the resulting higher power factor. Bergman and Levy^51^ theoretically predicted the possibility of higher thermoelectric power factors than the power factors of the pure components in a composite consisting of two phases, especially for a parallel slabs microstructure. They pointed out that the *zT* value of a composite can never be beyond the highest value of one of the constituent components^51^. An enhancement of both power factor and thermoelectric figure of merit beyond those of the components in a two-phase composite were first experimentally found in SiGe-Si nanocomposites^52^,^53^. This enhancement is mainly attributed to a higher carrier mobility, which in turn is interpreted to be induced by inhomogeneous doping only in Si nanograins instead of uniform doping in both components^52^. Selective doping will certainly contribute to the band engineering at the interface and may lead to charge transfer from one phase to the other one, which in turn can enhance *zT*^53^.

**Figure 8:**
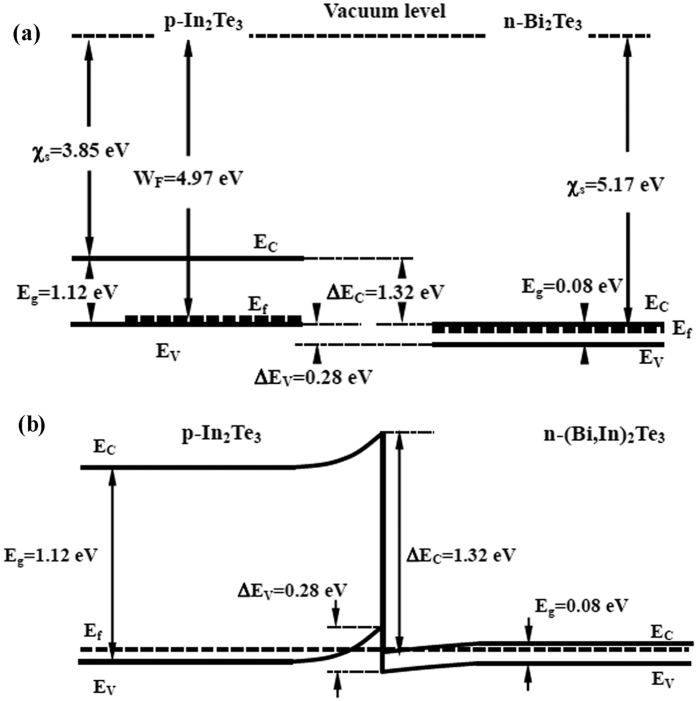
Schematic illustration of (**a**) band diagrams of Bi_2_Te_3_ and In_2_Te_3_ phase and (**b**) band bending at Bi_2_Te_3_/In_2_Te_3_ interface. E_g_: band gap; E_C_: conduction band; E_V_: valence band; E_f_: Fermi level; W_F_: working function; 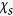: electron affinity; ΔE_C_: offset of conduction band; ΔE_V_: offset of valence band.

In the present work, the enhanced electrical conductivity (as a consequence of the higher carrier mobility, see Table 1) is the main reason for enhanced power factor and *zT*. As for SiGe-Si^52^,^53^, band alignment and charge transfer across Bi_2_Te_3_/In_2_Te_3_ interface was considered to be one of the possible reasons. A simple schematic illustration of the band diagram for an n-type (Bi,In)_2_Te_3_/p-In_2_Te_3_ interface is proposed in the present work, see Fig. 8, in which the maximum work function for In_2_Te_3_ and the minimum work function for Bi_2_Te_3_ are used. For p-type In_2_Te_3_, an electron affinity of 3.85 eV and a band gap of ∼1.12 eV have been reported^54^. It is not straightforward to determine the Fermi level of In_2_Te_3_ in BTIT in the present work. In view of the p-type behavior, we assume a Fermi level close to the valence band. Then the work function (W_F_) for In_2_Te_3_ ranges from 4.41 to 4.97 eV. As to n-type Bi_2_Te_3_ with 3 at% In, so far the band structure is not known and cannot be determined on the basis of the results in our present work. The recently reported band gap value, 0.08 eV, for Bi_2_Te_3_ with 4 at% In^55^ was used in the present work. We assumed a same electron affinity (5.17 eV^56^) as Bi_2_Te_3_. Considering the n-type transport behavior of (Bi,In)_2_Te_3_, a Fermi level at the same position of the conduction band is used. Even though the Fermi level of In_2_Te_3_ is higher than that of (Bi,In)_2_Te_3_ at the (Bi,In)_2_Te_3_/In_2_Te_3_ interface, the electrons tend to flow from In_2_Te_3_ to (Bi,In)_2_Te_3_ to balance the Fermi level at the contact, which in turns yields band bending. (Bi,In)_2_Te_3_ then can be expected to act as electron acceptor, and the formation of an electron accumulation layer close to the interface to In_2_Te_3_ can be safely assumed.

The Fermi levels of the two phases and the band offsets are crucial in the band alignment at the interface and the resulting charge transfer between the two phases. Experimentally, alloying/doping in each phase can induce a change of the Fermi level of each phase, and a change in the resulting band offset between the two phases. Moreover, thinking of the difference in the crystal structures of two phases, the lattice misfit and the resulting strain at the two-phase interface can affect the band alignment and then resulting charge transfer across the interface. These factors will lead to different band alignments and final properties^53^. Only suitable doping can lead to good band alignment to ensure the desired charge transfer from one phase to another^53^,^57^, which is an opportunity for enhanced *zT* values.

In the present case, electron transfer across the BT/IT interface could lead to a change in the thermoelectric properties of the two phases, similarly as for the SiGe-Si nanocomposites^52^,^53^. Calculations with the properties of the single separated phases (Bi,In)_2_Te_3_ and In_2_Te_3_ would necessarily lead to a deviation from the experimental data. With a modified charge distribution in the two components and thus a different electrical resistance of each phase, the enhancement of the conductivity of BTIT could probably be explained using the model in ref. 52. Our own simulation calculations suggest an accumulation layer that can also be regarded as a highly conductive third phase. Then, a three-phase model is more realistic for predicting the final properties. In this case, the highly conductive third phase leads to the increasing electrical conductivity with increasing Bi_2_Te_3_/In_2_Te_3_ interface density.

Band alignment at hetero-interfaces is regarded as one of the main reasons for enhanced electrical conductivity in two-phase nanocomposites, for example SiGe-Si^52^,^53^ and ZnO:Al – ZnS thin films^58^. As shown in Fig. 8, band bending at the Bi_2_Te_3_/In_2_Te_3_ interface may shift the Fermi level closer to the conduction band or even below the Fermi level (metal-like behavior). Such a metal-like surface state of Bi_2_Te_3_ may also be a possible reason for the increase of electrical conductivity with increasing BT/IT interface density. Together with the metal-like behavior at the BT/IT interfaces, a metal-like surface state of Bi_2_Te_3_ as a prominent example for a topological insulator^59^,^60^ may be another possible reason for the increase of electrical conductivity with increasing BT/IT interface density. Besides, In_2_Te_3_ was reported to exhibit a high room temperature charge carrier mobility, particularly a hole mobility of 1820 cm^2^V^−1^ s^−1^ and electron mobility of 2890 cm^2^V^−1^ s^−1^. Previous work on Cu_2_Se-CuAgSe^61^ indicates that the carrier mobility in each constituent phase plays a critical role for the electrical conductivity of the composite, especially when there is a big difference in the carrier mobility values between the phases.

Tentative Density Functional Theory (DFT) calculations with VASP (see e.g. ref. 62,63) using the Perdew-Burke-Ernzerhof^64^ exchange-correlation functional for a supercell built to reproduce the coherent BT/IT interface also predicted the possibility of a metal-like interface state. A crystallographic orientation between Bi_2_Te_3_ and In_2_Te_3_ of 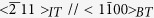 and 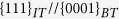
65 and an adjusted band off-set as in Fig. 8 were used. Metallic states localized on the Te atoms and on the neighboring In and Bi planes directly at the interface were observed. Generally, an extremely high carrier mobility in the vicinity of the interface is expected. The metal-like layer in the present work extends over a few atomic layers, which is not straightforward to detect experimentally. Metal-like conductivity originating from band alignment and charge transfer through the interface has been found in other systems, for example in LaAlO_3_/SrTiO_3_^66^ and SnO/SnO_2_^67^. It is worth noting that the band alignment and the resulting formation of a metallic layer at the BT/IT interface is closely related to the band offset between the two phases. The band offset in turn is influenced by alloying/doping and strain in the two phases. These effects are not considered in our reasoning on band alignment and the DFT calculations. However, also in the simplified form they demonstrate the plausibility of the proposed mechanisms.

## Conclusions

Metallurgical production methods exhibit high potential for generating high quality thermoelectric materials. In the present work, seeding zone melting and solid state precipitation were applied to produce n-type Bi_2_Te_3_-In_2_Te_3_ layered composites of pronounced anisotropy in structure and thermoelectric properties. The spacing of the Bi_2_Te_3_/In_2_Te_3_ layers and density of Bi_2_Te_3_/In_2_Te_3_ interfaces was tuned via the initial composition of the solid solution samples. Correlations between thermoelectric performance and microstructure were established. With increasing interface density, an increase in electrical conductivity and a decrease of bipolar transport properties lead to a substantial enhancement of *zT*. The enhanced electrical conductivity is likely to be due to band alignment and charge transfer across Bi_2_Te_3_/In_2_Te_3_ interface. The experimental strategy is promising to lead to pronounced high *zT* values, if the effect of the interfaces is exploited in combination with doping.

## Additional Information

**How to cite this article**: Liu, D. *et al*. Anisotropic layered Bi_2_Te_3_-In_2_Te_3_ composites: control of interface density for tuning of thermoelectric properties. *Sci. Rep.* **7**, 43611; doi: 10.1038/srep43611 (2017).

**Publisher's note:** Springer Nature remains neutral with regard to jurisdictional claims in published maps and institutional affiliations.

## Figures and Tables

**Table 1:** Carrier concentration (*n*) and mobility (*μ*) of the samples at room temperature (295 K).

Sample	R_H_ (10^−6^ m^3^A^−1^s^−1^)	*σ* (10^3^ S m^−1^)	*n* (10^19^ cm^−3^)	*μ* (cm^2^V^−1^ s^−1^)
BT-3In	−0.52	33	1.20	172
BTIT-4In	−0.59	62.5	1.12	368
BTIT-6In	−0.63	92	1.0	580
BTIT-7.5In	−0.55	175	1.13	963
